# The Association Between Energy Balance and Body Composition Changes in Recruits During Marine Corps Basic Training

**DOI:** 10.3390/nu18203363

**Published:** 2026-10-14

**Authors:** Jordan Beall, Andrea Givens, Thi Ngo, Emily Kloss, Brenda Niederberger, Laura Palombo, Daniel Bennett, Karen R. Kelly

**Affiliations:** 1 School of Exercise and Nutritional Sciences, San Diego State University, 5500 Campanile Drive, San Diego, CA 92182, USA; 2 Warfighter Performance Department, Naval Health Research Center, 140 Sylvester Road, San Diego, CA 92106, USA; andrea.c.givens2.ctr@health.mil (A.G.); emily.b.kloss.ctr@health.mil (E.K.); brenda.a.niederberger.ctr@health.mil (B.N.); laura.j.palombo.ctr@health.mil (L.P.); daniel.w.bennett32.ctr@health.mil (D.B.); 3 Leidos, Inc., 10260 Campus Point Drive, San Diego, CA 92121, USA

**Keywords:** energy balance, body composition, military nutrition, Marine Corps recruits, basic training, sex differences

## Abstract

**Background/Objectives**: United States Marine Corps (USMC) recruit training (“boot camp”) is a physically demanding environment in which recruits often experience significant changes in body composition. Energy balance (EB) plays a critical role in shaping these outcomes, yet how EB is associated with longitudinal fat and muscle changes remains unclear. This study evaluated the relationship between periodic EB and changes in body composition in male and female recruits. **Methods**: One hundred eighty-two USMC recruits (122 females, 60 males) volunteered; 158 recruits (106 females, 52 males) were included in the analysis. Energy intake was assessed on 10 distinct sampling days, energy expenditure was tracked via wearable devices, and body composition was obtained using bioelectrical impedance analysis. Statistical analyses included *t*-tests to compare sex differences in EB and general linear models to assess associations between EB, sex, and changes in body composition. **Results**: Across seven sampled days, females experienced periods of greater (more negative) daily energy deficit (−649 ± 775 kcal/day, 17%) compared to males (−388 ± 673 kcal/day, 8%, bootstrap *p* = 0.035). More negative estimated EB was associated with greater fat mass loss, with no evidence that this association differed by sex. In the 7-day model, the association between estimated EB and skeletal muscle mass differed by sex (sex × EB interaction, bootstrap *p* = 0.015); this interaction was not significant in the 10-day model that included the Crucible (bootstrap *p* = 0.792). **Conclusions**: USMC recruits experienced periods of low-moderate energy deficits during boot camp. Estimated EB during sampled training days was associated with fat mass change, whereas the association with skeletal muscle mass change differed by sex only in the 7-day model.

## 1. Introduction

The United States Marine Corps (USMC) requires recruits to undergo entry-level training, commonly referred to as boot camp. This rigorous training encompasses a variety of physically challenging tasks, all while upholding performance standards. Recruits face significant physical demands from the structured physical training outlined in the program of instruction, in addition to cumulative daily movements around the base.

Energy intake (EI) may not always sufficiently compensate for the increase in energy expenditure (EE), which could result in a negative energy balance (EB), a condition observed in military basic training settings [1,2,3]. The sudden escalation in physical activity, coupled with the stress of adapting to a new and highly regimented environment, may further contribute to this imbalance.

The negative EB observed during military training can lead to significant changes in body composition, including loss of both fat mass (FM) and fat-free mass (FFM; i.e., muscle). This decrease in body mass, particularly FFM, has been associated with declines in physical performance. Research has shown that performance in physically demanding tasks can decrease as a result of body mass loss, particularly when FFM is compromised [4,5]. However, studies examining these changes have produced mixed results. Some research has reported a decrease in both FM and FFM [3]. In contrast, other studies have found a loss in FM accompanied by a gain in muscle mass [6,7]. These discrepancies highlight the need for further investigation into how EB during boot camp is associated with body composition changes.

Biological sex may further influence the relationship between EB and body-composition change during military training. Female and male recruits differ on average in body size, baseline fat mass and skeletal muscle mass (SMM), and absolute energy expenditure; these differences may affect both the magnitude of estimated energy deficit and the physiological response to a given training stimulus [8]. In addition, prior military-training studies have reported sex-related differences in changes in fat-free mass, muscle mass, and substrate utilization during prolonged physical demand [9,10]. However, evidence is mixed and observed sex differences may depend on the training environment, baseline characteristics, energy availability, and the duration and severity of the energy deficit [11]. Therefore, whether sex modifies the association between estimated EB and changes in FM or SMM during USMC recruit training remains unclear.

While energy requirements and body composition changes during military training have been separately documented, relatively few studies have evaluated whether estimated EB during boot camp is associated with individual changes in FM and SMM, particularly in cohorts including substantial representation of females. Thus, the aims of this study were to (1) examine the association between estimated EB during sampled days of USMC boot camp and changes in body composition from baseline to post-training, and (2) evaluate whether the associations differed by sex. We hypothesized that more negative estimated EB would be associated with greater FM loss and less favorable SMM change. Because the direction and magnitude of sex differences in these associations are not established in USMC boot camp, the sex interaction analyses were considered exploratory.

## 2. Materials and Methods

### 2.1. Participants

As part of a larger research program examining musculoskeletal injuries (MSKIs) in young Marines, a total of 182 USMC recruits (*n* = 122 females; 60 males) attending boot camp at Marine Corps Recruit Depot in San Diego between March and November 2024 volunteered to participate in this study after providing written informed consent. Recruits were briefed by researchers in an auditorium without leadership present to ensure voluntary participation was not influenced by military staff. Recruits represented two different training battalions across three different training cycles, attending boot camp from either March to May 2024, June to August 2024, or September to November 2024. No separate *a priori* sample size calculation was performed for the present analysis of estimated EB and body composition changes. The sample was drawn from participants enrolled in the larger research program, with sample sizes determined by the availability of measurements. The study protocol (NHRC.2020.0008) was approved by the Naval Health Research Center Institutional Review Board and complied with all applicable federal regulations governing the protection of human subjects. Menstrual cycle status, urinary reproductive hormone metabolites, and salivary stress biomarkers were collected in female recruits as part of the larger research program and have been reported separately [12]. These measures were not included as predictors or effect modifiers in the present analysis of estimated EB and body composition change.

### 2.2. Energy Balance

EB was calculated as the difference between EI and EE measured on 10 training days throughout boot camp. Sampled days were the same training day across all three cohorts; similar in training events and meal opportunities. Seven sampled days occurred during routine training (weeks 1, 4, 7, and 10), while the final three sequential days captured “The Crucible” at the conclusion of boot camp. Accordingly, the mean EB for the 10 sampled days represents estimated EB across sampled routine training and Crucible days, not cumulative EB over the full 10-week training cycle. Percent energy deficit was calculated as [(EE − EI) ÷ EE] × 100. Daily EI was determined using the digital food photography method, which has been detailed by McClung and colleagues [13]. Researchers visited dining facilities on Tuesdays and Thursdays every 3 weeks throughout boot camp (weeks 1, 4, 7, and 10). Sampling days aligned with specific training phases and the dining facility’s rotating cycle menu. This repeating menu design was intentional to minimize the potential for food preferences to affect EI. Researchers photographed recruits’ meal trays before and after breakfast, lunch, and dinner. Later, photos were analyzed by Registered Dietitians and Nutritionists to quantify consumption. For evening snack packs that were issued to recruits on selected training days (weeks 4 and 7), all wrappers and packaging were retained, and the amount consumed was estimated and recorded from the returned packaging. Portions of all meals and snacks eaten were entered into Food Processor software (version 11.14, Trustwell, Beaverton, OR, USA) and nutrient data for menu items were determined. For field training days (weeks 7 and 10), recruits consumed Meal, Ready-to-Eat (MRE) combat rations away from the primary dining facility. All empty and partial MRE packaging was collected and preserved. Researchers analyzed the packaging to quantify percentage consumed per item before entry into the dietary software. Total daily EI was calculated from recorded scheduled meals, issued snack packs, and combat rations consumed by each recruit. Foods or beverages obtained through unrecorded sources, including food trading, could not be captured. As part of the larger MSKI research program, a more in-depth analysis of nutrient intake is in progress and will be reported in a subsequent publication.

### 2.3. Energy Expenditure

All participants were provided Polar Grit X wrist-worn physiological monitors (Polar Electro, Kempele, Finland), based on prior validation for EE in a military population [14]. Participants were instructed to wear the devices continuously for 10 weeks of boot camp, except for a 4 h window each week during which research staff retrieved the devices for charging and data transfer. EE was obtained directly from Polar’s proprietary algorithm, which used heart rate data and participant characteristics, e.g., age, sex, height, weight that had been programmed into each individual device. The device output was used as provided; the Polar system did not provide conventional wear time or non-wear time classifications. Instead, it reported proprietary “active time”, defined by the manufacturer as time spent on feet or on the move. Data were cleaned only to include days in which participants accrued at least 4 h of “active time”. This threshold was selected to accommodate variability in training intensity, weekend “off” days, and periods of light duty that were considered representative of recruit training conditions. Because non-wear time could not be directly determined, the 4 h daily active time threshold served as a proxy to define usable data. EE was not extrapolated or imputed for periods without recorded device data. Thus, EB estimates reflect observed device derived expenditure paired with EI data on days meeting the prespecified criterion. A valid EB day was defined as a day with matched EE and EI data. At least one valid EB day was required for participant inclusion in the unrestricted analyses; no higher minimum was imposed. All available valid days within each sampling period were used to calculate each participant’s EB estimate.

### 2.4. Body Composition

Pre- and post-body composition measurements were taken in week 1 and week 10 to evaluate changes over the course of the study period. Participants’ body fat percentage and skeletal muscle mass (SMM) in kilograms were assessed using the InBody 770 (Seoul, Republic of Korea), a multi-frequency bioelectrical impedance analysis (BIA) device that the USMC has adopted for use as directed by the Training and Education Command. To obtain FM in kilograms, total body mass was multiplied by body fat percentage, then divided by 100. For the InBody measurements, participants wore the same lightweight uniform and performed the assessment at the same time of day under the same conditions: in the morning after emptying bowel and bladder. As part of the larger MSKI research program, detailed analysis of body composition changes across boot camp has been published elsewhere [15]. The prior report did not evaluate associations between EB and individual changes in FM or SMM. The present analysis therefore focuses on EB association; sex stratified pre–post body composition values are provided descriptively in the Supplementary Material.

### 2.5. Statistical Analysis

Participant baseline demographics (age, height, weight, and Body Mass Index (BMI)) were stratified by sex and compared using independent samples *t*-tests. Equality of variances was verified via Levene’s test. Where the assumption of equal variances was violated, Welch–Satterthwaite adjustments to the degrees of freedom were applied.

To characterize the amount of available data, median, interquartile range (IQR), full range (min–max) and categorical distribution of valid EB days were determined per participant overall and by sex and training cohort. Additionally, exploratory comparisons of baseline characteristics and body composition changes across valid day categories were evaluated using one-way ANOVA.

An average daily EB was computed for each recruit for two tracking periods: 7 days of standard training, and 10 days (7 days of standard training plus 3 days of the Crucible). Because the final 3-day operational block, “The Crucible” represented an intentional, extreme energy deficit for recruits’ final culminating event, both sampling periods were evaluated as complementary exploratory analyses for each body composition outcome. Potential sex differences in EB across both time frames were evaluated using independent samples *t*-tests.

Data normality within sexes was evaluated using the Shapiro–Wilk test. Due to observed non-normal distributions in daily EB and changes in fat mass (*p* < 0.05), primary hypotheses evaluating changes in fat mass (ΔFM), and change in SMM (ΔSMM), were assessed using General Linear Models (GLM) coupled with non-parametric bias-corrected accelerated (BCa) bootstrapping (2000 resamples) to generate robust parameter estimates and 95% confidence intervals (CIs).

Separate GLMs were fitted for each combination of body composition outcome (ΔFM and ΔSMM) and EB assessment days (7-day and 10-day), yielding four models. In each model, the dependent variable was ΔFM or ΔSMM; the primary continuous predictor was participant level mean daily EB calculated over the corresponding 7-day or 10-day sampled days. Sex was included as a binary categorical factor (male = 0, female = 1). A sex × EB interaction term was initially included in all models. Additional FM models without the interaction term were fitted to estimate a common adjusted association across sexes, while the SMM models retained the interaction term. All models were adjusted for training cohort (1, 2, or 3), baseline body weight, and the number of valid EB days within the respective assessment period. Female was the reference category in the GLMs.

Training cohort was modeled as a three-level categorical covariate to account for the organizational structure of data collection. Each cohort represented a unique combination of battalion, company, training cycle, and calendar/season; consequently, independent effects of these nested factors could not be estimated separately. Sex was represented in all cohorts, although distribution varied by cohort.

GLMs used complete cases for the outcome and all included predictors and covariates. One participant lacked baseline body weight and was therefore excluded from all adjusted models, yielding a model sample of 157 participants.

Post hoc sensitivity analyses replaced baseline body weight with baseline FM in the ΔFM models and baseline SMM in the ΔSMM models, using the same 157 participants as the main models to maintain a consistent comparison sample. Separate sensitivity analyses repeated the baseline body weight adjusted models after excluding participants with two or fewer valid EB days within the corresponding sampling days; sample sizes for these restricted analyses are reported.

Two-tailed statistical significance was established *a priori* at *p* < 0.05. All statistical analyses were conducted using IBM SPSS Statistics version 29.0.1.1 (IBM Corp., Armonk, NY, USA).

## 3. Results

### 3.1. Participants

Recruit data were included in the results if they had pre- and post-body composition measurements and at least 1 day of EB data. A total of 24 recruits were excluded from data analysis: 2 recruits using hormonal contraception were excluded before study measurements as part of the larger research program exclusion criteria; 19 recruits were lost to attrition from boot camp (due to injury, failure to meet standards, or personal choice); one recruit did not have any EE data; one recruit did not have any meal photographs; and one recruit did not have a post-body composition measurement. The overall sample included 158 recruits (*n* = 106 females, 52 males). The adjusted FM and SMM models included 157 recruits (*n* = 105 females, 52 males), because one female participant lacked baseline body weight, which was included as a covariate in all models. Her available data were retained in descriptive and other analyses where required measurements were complete. Demographics are presented in Table 1. Males were taller and weighed more than females at baseline (both *p* < 0.001), however age and BMI did not differ by sex (age, *p* = 0.175; BMI, *p* = 0.691).

Participants were enrolled in three sequential training cohorts. Both males and females were represented in each cohort, although the proportion of male and female participants varied (Table 2).

Valid days of EB data are shown distributed by sex and training cohort in Table 3. In exploratory comparisons, baseline body composition measures and changes in FM and SMM were not different across valid day categories (Supplementary Table S1); the single participant with 1 valid day was retained descriptively but excluded from inferential comparisons across valid day categories; this exclusion was separate from the complete case exclusion applied to the GLMs. Supplementary Table S2 provides sex-stratified body composition changes presented descriptively to provide context for the present energy balance analyses. Detailed pre-post body composition analyses from this recruit cohort have been reported previously by Kloss et al. [15].

### 3.2. Energy Balance

The training events and meal types of the 10 intermittent training days that EB was assessed on are shown in Supplementary Table S3. Comparing the average EB of the 7-day sampled days by sex revealed that females experienced significantly greater (more negative) periods of daily energy deficit compared to males (females: −649 ± 775 kcal/day vs. males: −388 ± 673 kcal/day; *t*(115.30) = 2.18, bootstrap *p* = 0.035, 95% BCa CI: 27.60, 499.97). This corresponded to a relative energy deficit of 17% for females, and 8% for males. Across the 7-day period, 11 males (21%) and 43 females (41%) had an individual energy deficit percentage that exceeded 20%.

Conversely, including the Crucible to average EB for all 10 sampled days, no significant sex differences were observed. The inclusion of this substantial negative EB resulted in a severe energy deficit for the 10-day period that did not differ by sex (males: −1466 ± 959 kcal/day; females: −1417 ± 719 kcal/day; *t*(156) = −0.36, bootstrap *p* = 0.748, 95% BCa CI: −352.96, 234.94). This corresponded to a relative energy deficit of 34% for females, and 26% for males. Across the 10-day period, 36 males (69%) and 85 females (80%) had an individual energy deficit percentage that exceeded 20%. To evaluate the potential impact of low data yield on these outcomes, a post hoc sensitivity filter was performed by dropping all participants with low data yield (≤2 valid days of EB data). The restricted analysis confirmed stability across models both including the Crucible (*t*(150) = −0.17, bootstrap *p* = 0.862) and excluding the Crucible (*t*(115.12) = 2.05, bootstrap *p* = 0.039).

### 3.3. Association Between Energy Balance and Change in Fat Mass

Complete multivariable regression results for ΔFM across the 7-day and 10-day sampled days are presented in Table 4. Both models included ΔFM as the dependent variable and were adjusted for sex, cohort, baseline body weight (kg), valid EB days for the corresponding sampling days, and EB for the corresponding sampling days. To evaluate whether the association between estimated EB and ΔFM differed by sex, sex × EB interactions were first tested in otherwise identical models. The interaction was not significant in either the 10-day model including the Crucible (bootstrap *p* = 0.131) or the 7-day model excluding the Crucible (bootstrap *p* = 0.851). Therefore, common slope models without the interaction term were fitted to estimate the adjusted association between estimated EB and ΔFM across sexes.

**Figure 1:**
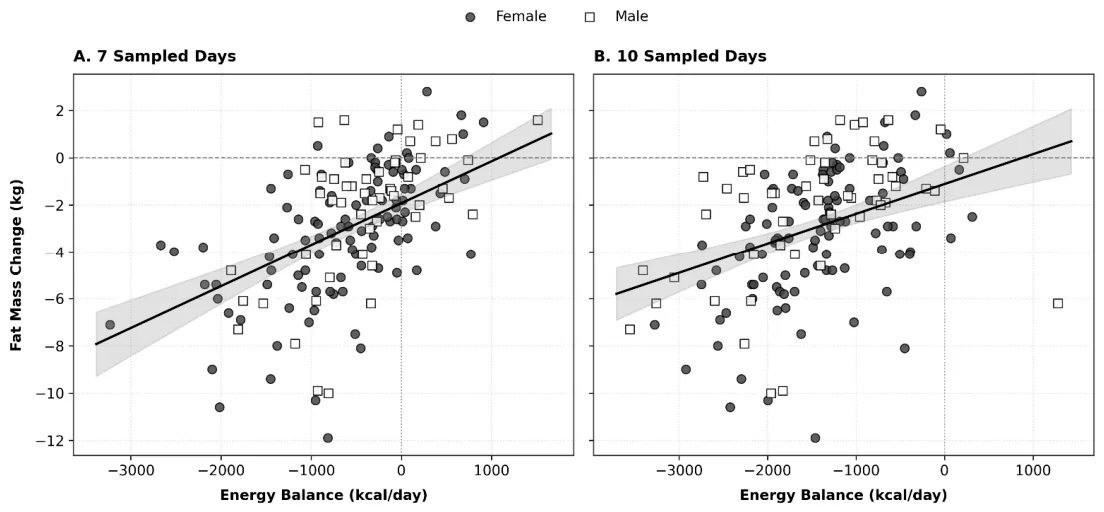
Association between estimated energy balance during sampled training days and change in fat mass (kg) among USMC recruits during boot camp. *Note.* Points represent individual participants; solid lines show predictions from the common slope GLMs, and shading represents 95% confidence intervals. Models included baseline body weight (kg), cohort, sex, estimated EB, and valid energy balance days.

In the 10-day model, the overall model was significant and accounted for 40.4% of the variance in ΔFM during boot camp, *R*^2^ = 0.427; adjusted *R*^2^ = 0.404; *F*(6, 150) = 18.62, *p* = <0.001. Estimated EB was positively associated with change in FM (*B* = 0.00126, *t* = 5.317, bootstrap *p* < 0.001, 95% BCa CI: 0.001, 0.002), indicating that periods of lower (more negative) EB values were associated with greater reductions in FM. Scaled to standard dietary metrics, a 500 kcal/day lower estimated EB corresponded to approximately 0.63 kg greater decrease in FM from baseline to post-training. The 7-day model showed a similar positive association between estimated EB and FM change (*B* = 0.00178, *t* = 7.467, BCa 95% CI: 0.001, 0.002, bootstrap *p* < 0.001; Table 4). Figure 1 displays individual observed values and covariate adjusted regression predictions for the association between estimated EB and change in FM. A lower estimated EB was associated with greater loss of FM.

### 3.4. Association Between Energy Balance and Change in Skeletal Muscle Mass

Complete multivariable regression results for ΔSMM across the 7-day and 10-day sampled days are presented in Table 5. Both models included ΔSMM as the dependent variable and were adjusted for sex, cohort, baseline body weight (kg), valid EB days for the corresponding sampling days, EB, and the sex × EB interaction.

**Figure 2:**
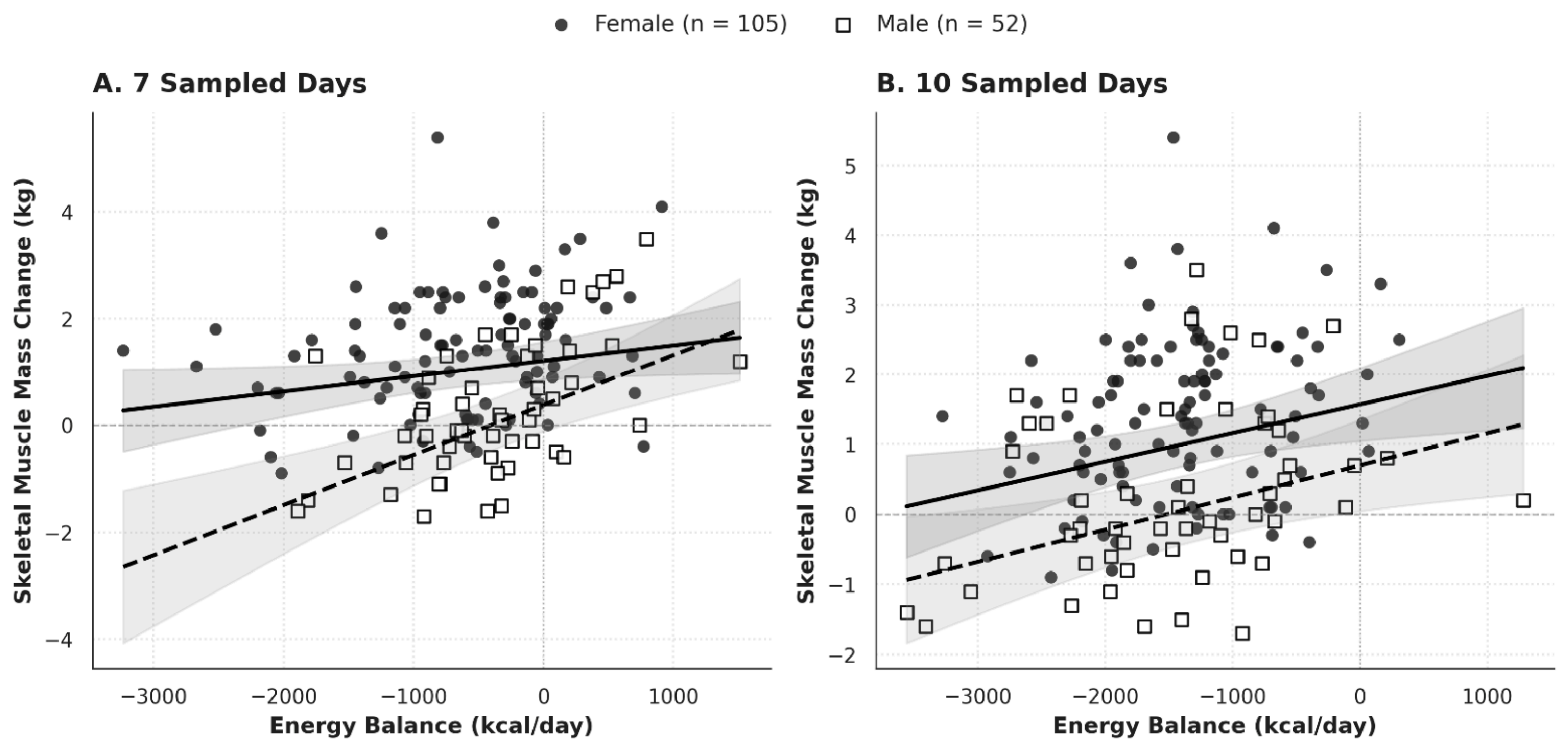
Association between estimated energy balance during sampled training days and change in skeletal muscle mass (kg) by sex among USMC recruits during boot camp. *Note.* Points represent individual participants; solid lines represent adjusted predictions for females, and the dashed lines represent adjusted predictions for males; shading represents 95% confidence intervals. Predictions were derived from general linear models including sex, estimated energy balance, the sex × energy balance interaction, cohort, baseline body weight (kg), and valid energy balance days. (**A**) shows the 7-sampled days excluding the Crucible; (**B**) shows all 10 sampled days, including the Crucible. Skeletal muscle mass change was calculated as post-training minus baseline; positive values indicate gains.

In the 7-day model, the overall model was statistically significant and accounted for 33.0% of the variance in ΔSMM during boot camp, *R*^2^ = 0.360; adjusted *R*^2^ = 0.330; *F*(7, 149) = 11.97, *p* = < 0.001. The association between estimated EB and ΔSMM differed by sex (male × EB interaction: *B* = 0.00065; BCa 95% CI: 0.0001, 0.0010, bootstrap *p* = 0.015). With females as the reference category, the positive male × EB interaction coefficient indicated a more positive modeled slope among males; this interaction was not evident in the 10-day model that included the Crucible. Figure 2 illustrates the covariate adjusted sex-specific model predictions for the association between estimated EB and change in SMM.

Sex-stratified adjusted models are reported in Supplementary Table S4. Estimated EB was positively associated with change in SMM among males (*B* = 0.000248; BCa 95% CI: 0.000001, 0.001; bootstrap *p* = 0.033), whereas the within-female association was not significant (*B* = 0.000119; BCa 95% CI: −0.000019, 0.001; bootstrap *p* = 0.285). The pooled sex × EB interaction remained the formal test of differences in slopes between sexes.

### 3.5. Sensitivity Analyses

In post hoc sensitivity analyses replacing baseline body weight with the baseline value of the corresponding body composition outcome, estimated EB remained positively associated with ΔFM in both sampling periods (10-day: *B* = 0.00082, BCa 95% CI: 0.00042, 0.00132; 7-day: *B* = 0.001181, BCa 95% CI: 0.00076, 0.00157; both bootstrap *p* < 0.001). For ΔSMM, the sex × EB interaction remained significant in the 7-day model adjusted for baseline SMM (*B* = 0.00064, BCa 95% CI: 0.00007, 0.00122, bootstrap *p* = 0.019), but not in the 10-day model (bootstrap *p* = 0.994; Supplementary Table S5). These analyses used the same 157 participants as the baseline-body-weight-adjusted models.

After excluding participants with two or fewer valid EB days within the corresponding sampling day periods, complete-case samples comprised 129 participants for the 7-day models and 151 for the 10-day models. The positive association between estimated EB and ΔFM remained significant in both models (10-day: *B* = 0.00126, BCa 95% CI: 0.00074, 0.00182; 7-day: *B* = 0.001786, BCa 95% CI: 0.00127, 0.00233; both bootstrap *p* < 0.001). The 7-day sex × EB interaction for ΔSMM had a similar point estimate to the unrestricted model but a wider confidence interval that included zero (*B* = 0.00065, BCa 95% CI: −0.000034, 0.00139, bootstrap *p* = 0.066). The interaction remained nonsignificant in the restricted 10-day model (bootstrap *p* = 0.835; Supplementary Table S6).

## 4. Discussion

This study examined associations between estimated EB during sampled training days and longitudinal body-composition changes among female and male USMC recruits during boot camp. During the seven sampled routine-training days, female recruits had more negative mean estimated EB than male recruits, corresponding to mean estimated energy deficits of 17% and 8%, respectively. More negative estimated EB was associated with greater reductions in FM, with no evidence that this association differed by sex. In contrast, the association between estimated EB and change in SMM differed by sex in the 7-day model, with a more positive modeled slope among male recruits. This sex × EB interaction was not significant in the 10-day model that included the Crucible period.

These results should be interpreted in the context of the sampling design. Estimated EB was derived from up to 10 intermittent assessment days rather than continuously across the full 10-week training cycle. The 7-day estimate reflects sampled routine training days, whereas the 10-day estimate also incorporates the Crucible, a distinct field-training context characterized by high workload, limited sleep opportunity, field rations, and more negative estimated EB. Thus, the observed SMM interaction was sensitive to the sampled exposure window and should not be interpreted as evidence of a general sex-specific association across all 10 weeks of boot camp.

To our knowledge, this is the first study to evaluate associations between estimated EB and body composition changes during USMC boot camp while including a large cohort of female recruits. The energy deficits observed during sampled days are consistent with previous reports from military training environments, where EE often exceeds EI because of high physical demands, limited eating time, and operational constraints [1,3,5,16]. The negative estimated EB observed in the present study was greater when the sampled days included the Crucible. From an operational perspective, the results support continued characterization of EE and EI across distinct training phases and evaluation of substantial energy deficits alongside operationally relevant outcomes including physical performance, injuries, and health.

Consistent with the overall negative estimated EB observed during sampled training days, greater energy deficits were associated with larger reductions in FM in both the 7-day and 10-day models. This finding aligns with previous research demonstrating that sustained energy deficits during military training promote reductions in FM [3,6,7]. However, because EI and EE were assessed intermittently in the present study, the findings should be interpreted as associations between sampled-day estimated EB and cumulative pre-to-post FM change. The sex × EB interaction was not significant in either the 7-day or 10-day model, providing no evidence that the association differed by sex. In the 10-day model, a 500 kcal/day lower estimated EB was associated with approximately 0.63 kg greater FM loss from baseline to post-training; this scaling should not be interpreted as a measured sustained 500 kcal/day deficit across the full training cycle. In a prior report from this cohort, 34% of recruits entered training outside of USMC body composition standards compared to 12% upon completion [15].

In contrast to FM, the association between EB and SMM change varied by sex in the 7-day model. In the pooled model, the positive male × EB interaction indicated a more positive modeled association between estimated EB and SMM change among males than females. This interaction was not observed in the 10-day model including the Crucible. The absence of an interaction in the 10-day model may reflect the different exposure context represented by the Crucible days, including field conditions, high physical demands, restricted sleep opportunity, limited food availability, and a more negative estimated EB. However, the present analysis cannot determine which feature of the Crucible accounted for the difference between the 7-day and the 10-day results. Because the two models represent different sampled days rather than repeated measures of a stable EB exposure, these findings should not be generalized to the full training cycle.

The sensitivity analyses also support a cautious interpretation. The sex difference in the 7-day EB-SMM association remained significant when baseline SMM replaced baseline body weight in the model. When participants with two or fewer valid EB days were excluded, the estimated difference between the male and female slopes was similar, but the result was no longer significant. These findings reinforce the exploratory nature of the observed sex differences.

The present interaction result does not indicate that male recruits gained more SMM overall. In the prior descriptive body composition analysis from this cohort that compared average group-level body composition changes, female recruits demonstrated larger mean pre-to-post SMM gains than male recruits [15]. The present analysis instead evaluated whether between-recruit variation in estimated EB during sampled days was associated with the magnitude of SMM change. As such, females may have had larger average SMM gains while the 7-day association between EB and change in SMM was more positive among male recruits. Sex-specific differences in lean mass or fat-free mass change have also been reported in USMC Officer Candidate School and Army Ranger training [11,17]. Female and male recruits may have different body composition changes despite similar training exposure [18].

Several factors could contribute to sex-related differences in the association between estimated EB and SMM change, including baseline body composition, habitual physical activity prior to boot camp, the relative novelty of the training stimulus, or endocrine responses. Menstrual function, urinary reproductive metabolites, and salivary stress biomarkers were assessed in female recruits participating in the same larger research program and are reported separately [12]. In that analysis, menstrual cycle disruption was common during recruit training: among recruits with analyzable menstrual cycle data, 53% self-reported irregular cycles during boot camp, and urinary hormone metabolite measures classified 92% as having menstrual cycle disruption. Most disruptions occurred during the first menstrual cycle of boot camp. However, menstrual status and hormonal measures were not evaluated as predictors or effect modifiers in the present models. These findings cannot establish whether endocrine or menstrual function changes contributed to the observed associations between estimated EB and SMM change. Further analyses integrating EB, reproductive hormone measures, stress biomarkers, and body composition changes are warranted.

A relative energy deficit exceeding 20% was used as a descriptive reference point because this magnitude is used in military performance nutrition contexts and provides an interpretable benchmark. Prior military literature indicates that greater cumulative negative EB may be associated with reductions in lower body strength and power and broader physical and cognitive effects, but the severity and duration of deficit matter [19,20]. The present study design was not intended to determine an EB threshold for adverse outcomes.

These findings have important practical implications for military nutrition and training. The observed associations between energy balance and changes in FM and SMM support the value of characterizing energy intake and expenditure across training phases. To aid interpretation, individual energy deficit percentage was calculated to provide context for the magnitude of the deficit; however, this is a descriptive reference point, not a validated threshold for safe or sustainable adaptation during training. The assessment window-dependent sex interaction for change in SMM also indicates that relationships between EB and body composition may differ across routine and high demand training periods. The findings identify estimated EB during sampled training days as a potential target for future prospective research examining whether nutrition strategies influence performance, body composition, health, or readiness outcomes.

The present study has several strengths. It had high ecological validity, capturing USMC recruits during 10 weeks of real-world basic training across 3 seasonally distinct cohorts. It was conducted within a highly structured military environment, reducing behavioral variability and increasing compliance with study procedures. EI was estimated via validated digital food photography rather than relying on participant self-report. Additionally, the inclusion of a large female cohort allowed for sex-based comparisons that remain underrepresented in military physiology research.

This study is not without limitations. The observational design limits causal inference between EB and body composition changes. EB was averaged using a periodic sampling design across 10 collection days and may not fully represent day-to-day variability throughout boot camp. EB averages from available days do not exclude larger individual day deficits. Recruits may have had access to food sources outside of the recorded meals and issued rations, including foods or beverages obtained through food trading or other sources. These items were not captured; therefore, estimated EI may underestimate total intake and overestimate energy deficit. The proprietary Polar algorithm did not provide conventional wear time and non-wear time classifications, and the 4 h active time does not ensure that a given day represents full 24 h energy expenditure. Although we retained lower activity days to reflect routine recruit training conditions and did not extrapolate expenditure for incomplete observations, exposure misclassification remains possible. Retaining participants with minimal data yield (e.g., a single day of EB data) increases the potential for exposure misclassification when relating sampled EB to a 10-week outcome. To address this, individual data yield was adjusted for in the analysis by utilizing valid tracking days as a continuous model covariate. However, this adjustment cannot eliminate measurement error or guarantee that EB estimates based on relatively few observed days represent participants’ usual energy balance. The distribution of valid days, including the number of participants with few valid days, should therefore be considered when interpreting the findings. A secondary post hoc sensitivity filter excluding participants with ≤2 valid days preserved the FM associations, while the 7-day SMM interaction had a similar point estimate but was not significant. These restrictions did not eliminate measurement error or representativeness concerns. Finally, body composition was assessed using BIA, which can be influenced by hydration status. To address this, measurements were standardized with respect to time of day and lightweight uniform; food and fluid intake as well as recent exercise could not be fully controlled in the recruit training environment.

## 5. Conclusions

During sampled USMC boot camp training days, more negative estimated EB was associated with greater FM loss, with no evidence that the association differed by sex. The association between estimated EB across 7 routine training days and change in SMM differed by sex, with a more positive modeled slope among male recruits; this exploratory finding was not observed in the 10-day model that included the Crucible. Because EB was assessed intermittently and body composition was estimated using BIA, these findings should be interpreted as observational associations within the sampled training days rather than evidence of cumulative 10-week EB effects or causal mechanisms. Future studies with more complete EB monitoring and including performance, health, and readiness outcomes are needed.

## Figures and Tables

**Table 1:** Participant Demographics.

	Females (*n* = 106)	Males (*n* = 52)
Age (years)	19.0 ± 1.7	18.7 ± 1.1
Height (cm)	161.3 ± 6.9	174.5 ± 7.2
Weight (kg)	60.7 ± 8.5	71.6 ± 10.6
Body mass index (kg/m^2^)	23.3 ± 3.0	23.5 ± 3.1

*Note*. Values represent mean ± standard deviation. Female baseline body weight and BMI are based on 105 participants; other sample sizes reflect available observations.

**Table 2:** Distribution of participants by sex and training cohort.

Training Cohort	Overall, *n* (%)	Males, *n* (%)	Females, *n* (%)
Cohort 1	39 (100.0%)	15 (38.5%)	24 (61.5%)
Cohort 2	62 (100.0%)	27 (43.5%)	35 (56.5%)
Cohort 3	57 (100.0%)	10 (17.5%)	47 (82.5%)

*Note.* Cohort 1: 3rd Battalion, Lima Company, March–May 2024; Cohort 2: 2nd Battalion, Golf Company, June–August 2024; Cohort 3: 2nd Battalion, Golf Company, September–November 2024.

**Table 3:** Distribution of valid energy balance days per participant overall, by sex, and by training cohort.

**Panel A. Overall and by Sex**			
**Valid Days**	**Overall, *n* (%)**	**Males, *n* (%)**	**Females, *n* (%)**
1 day	1 (0.6%)	0 (0.0%)	1 (0.9%)
2 days	5 (3.2%)	0 (0.0%)	5 (4.7%)
3–5 days	41 (25.9%)	15 (28.8%)	26 (24.5%)
6–8 days	92 (58.2%)	27 (51.9%)	65 (61.3%)
9–10 days	19 (12.0%)	10 (19.2%)	9 (8.5%)
Median (IQR)	7 (5–8)	7 (5–8)	7 (5–8)
Range (Min–Max)	1–10	3–10	1–10
**Panel B. By Training Cohort**			
**Valid Days**	**Cohort 1, *n* (%)**	**Cohort 2, *n* (%)**	**Cohort 3, *n* (%)**
1 day	0 (0.0%)	1 (1.6%)	0 (0.0%)
2 days	0 (0.0%)	4 (6.5%)	1 (1.8%)
3–5 days	9 (23.1%)	29 (46.8%)	3 (5.3%)
6–8 days	29 (74.4%)	23 (37.1%)	40 (70.2%)
9–10 days	1 (2.6%)	5 (8.1%)	13 (22.8%)
Median (IQR)	6 (6–7)	5 (4–7)	8 (7–8)
Range (Min–Max)	4–9	1–9	2–10

Note: IQR = Interquartile Range. Percentages are column percentages. A valid energy balance day was defined as at least 4 h of active time matching energy intake sampling days. Cohort 1: 3rd Battalion, Lima Company, March–May 2024; Cohort 2: 2nd Battalion, Golf Company, June–August 2024; Cohort 3: 2nd Battalion, Golf Company, September–November 2024.

**Table 4:** General Linear Model: Fat Mass Change.

**Panel A: Fat Mass Change (ΔFM)—10-Day Energy Balance**				
**Parameter**	**Unstandardized *B***	**SE**	**BCa 95% CI**	**Bootstrap *p*-Value**
Intercept	6.116	1.507	3.449, 9.012	0.001
Male sex (reference: female)	2.696	0.400	1.940, 3.484	<0.001
Cohort 1 (reference: Cohort 3)	−1.356	0.446	−2.339, −0.487	0.002
Cohort 2 (reference: Cohort 3)	−1.587	0.448	−2.493, −0.726	<0.001
Baseline body weight (kg)	−0.113	0.024	−0.159, −0.066	<0.001
10-Day Energy Balance	0.00126	0.0003	0.001, 0.002	<0.001
Valid EB Days (10-Day)	0.013	0.115	−0.226, 0.241	0.904
Model Fit: *n* = 157; *R*^2^ = 0.427; Adjusted *R*^2^ = 0.404; *F*(6, 150) = 18.622, *p* < 0.001				
**Panel B: Fat Mass Change (ΔFM)—7-Day Energy Balance**				
**Parameter**	**Unstandardized *B***	**SE**	**BCa 95% CI**	**Bootstrap *p*-Value**
Intercept	5.676	1.296	3.224, 8.427	<0.001
Male sex (reference: female)	2.146	0.376	1.406, 2.923	<0.001
Cohort 1 (reference: Cohort 3)	−2.022	0.434	−2.952, −1.165	<0.001
Cohort 2 (reference: Cohort 3)	−1.358	0.398	−2.141, −0.585	0.003
Baseline body weight (kg)	−0.100	0.021	−0.142, −0.060	<0.001
7-Day Energy Balance	0.00178	0.0002	0.001, 0.002	<0.001
Valid EB Days (7-Day)	−0.221	0.161	−0.587, 0.100	0.170
Model Fit: *n* = 157; *R*^2^ = 0.504; Adjusted *R*^2^ = 0.484; *F*(6, 150) = 25.389, *p* < 0.001				

*Note.* FM, fat mass; EB, estimated energy balance (kcal/day); CI, confidence interval; SE, standard error. The dependent variable was change in FM (kg) from baseline to post-training. Panel A represents the 10 sampled days (including the Crucible). Panel B presents the seven sampled days (excluding the Crucible). Both models were adjusted for sex, cohort, baseline body weight (kg), valid EB days for the corresponding sampling days, and EB for the corresponding sampling days. Models shown do not include the sex × EB interaction term and therefore estimate the common adjusted association between estimated EB and fat mass change across sexes. Sex × EB interactions were tested in otherwise identical models and were not significant for either the 10-day model (bootstrap *p* = 0.131) or the 7-day model (bootstrap *p* = 0.851); interaction terms were omitted from the common slope models presented here. Unstandardized coefficients (*B*) are estimates from the fitted GLMs. Bootstrap standard errors (SE) and two-sided *p*-values were obtained using 2000 nonparametric resamples, and 95% CIs were calculated using the bias corrected and accelerated (BCa) method. Biological sex was coded as male = 0 and female = 1 in the dataset. In the model, female was specified as the reference category.

**Table 5:** General Linear Model: Skeletal Muscle Mass change.

**Panel A: Skeletal Muscle Mass Change (ΔSMM)—7-Day Energy**				
**Parameter**	**Unstandardized *B***	**SE**	**BCa 95% CI**	**Bootstrap *p*-Value**
Intercept	2.436	0.586	1.318, 3.456	<0.001
Male sex (reference: female)	−0.829	0.239	−1.287, −0.395	0.002
Cohort 1 (reference: Cohort 3)	0.680	0.235	0.240, 1.134	0.002
Cohort 2 (reference: Cohort 3)	0.374	0.201	−0.044, 0.779	0.062
Baseline Body Weight (kg)	−0.025	0.008	−0.042, −0.006	0.007
7-Day Energy Balance	0.00029	0.0003	−0.00002, 0.001	0.072
Valid EB Days (7-Day)	0.093	0.081	−0.056, 0.254	0.255
Male sex × 7-Day Energy Balance	0.00065	0.0004	0.0001, 0.0010	0.015
Model Fit: *n* = 157; *R*^2^: 0.360; Adjusted *R*^2^: 0.330; *F*(7, 149) = 11.97, *p* < 0.001				
**Model: Skeletal Muscle Mass Change (ΔSMM)—10-Day Energy Balance**				
**Parameter**	**Unstandardized *B***	**SE**	**BCa 95% CI**	**Bootstrap *p*-Value**
Intercept	2.694	0.623	1.484, 3.858	<0.001
Male sex (reference: female)	−0.873	0.349	−1.545, −0.180	0.016
Cohort 1 (reference: Cohort 3)	0.811	0.247	0.309, 1.312	0.002
Cohort 2 (reference: Cohort 3)	0.337	0.217	−0.084, 0.774	0.132
Baseline Body Weight (kg)	−0.028	0.009	−0.045, −0.009	0.002
10-Day Energy Balance	0.00041	0.0002	0.0001, 0.0007	0.011
Valid Tracking Days (10-Day)	0.107	0.053	0.005, 0.208	0.042
Male sex × 10-Day Energy Balance	0.00005	0.0003	−0.0006, 0.0006	0.792
Model Fit: *n* = 157; *R*^2^: 0.326; Adjusted *R*^2^: 0.295; *F*(7, 149) = 10.306, *p* < 0.001				

*Note.* SMM, skeletal muscle mass; EB, estimated energy balance (kcal/day); CI, confidence interval; SE, standard error. The dependent variable was change in SMM (kg) from baseline to post-training. Panel A represents the 7 sampled days (excluding the Crucible). Panel B presents the 10 sampled days (Including the Crucible). Both models were adjusted for sex, cohort, baseline body weight (kg), valid EB days for the corresponding sampling days, EB, and the sex × EB interaction. Unstandardized coefficients (*B*) are estimates from the fitted GLMs. Bootstrap standard errors (SE) and two-sided *p*-values were obtained using 2000 nonparametric resamples, and 95% CIs were calculated using the bias corrected and accelerated (BCa) method. Biological sex was coded as male = 0 and female = 1 in the dataset. In the model, female was specified as the reference category. The EB coefficient is the female specific slope, and the male × EB term is the male–female difference in EB slopes.

## Data Availability

The datasets generated and/or analyzed during the current study are not publicly available due to personally identifiable information regulations, but they may be made available by the corresponding author on reasonable request and approval by the Naval Health Research Center Institutional Review Board/Privacy Office.

## Supplementary Materials

The following supporting information can be downloaded at https://www.mdpi.com/article/10.3390/nu18203363/s1, Table S1: Baseline characteristics and body composition changes by number of valid energy balance days; Table S2: Pre- and Post-Training Body Composition Changes Stratified by Sex; Table S3: Program of Instruction for Energy Balance Assessment Days; Table S4: Sex-stratified adjusted associations between estimated energy balance during sampled days and change in skeletal muscle mass; Table S5. Summary of sensitivity analyses replacing baseline body weight with baseline fat mass or skeletal muscle mass; Table S6. Summary of sensitivity analyses excluding participants with two or fewer valid energy balance days within the corresponding sample day window.

## Disclaimer

K.R.K. is an employee of the U.S. Government. This work was prepared as part of her official duties. Title 17, U.S.C. §105 provides that copyright protection under this title is not available for any work of the U.S. Government. Title 17, U.S.C. §101 defines a U.S. Government work as work prepared by a military service member or employee of the U.S. Government as part of that person’s official duties. Report No. 26-64 was supported by the Defense Health Agency under work unit no. N1627 project MO230125. The views expressed in this article are those of the authors and do not necessarily reflect the official policy or position of the Department of the Navy, Department of Defense, nor the U.S. Government. The study protocol was approved by the Naval Health Research Center Institutional Review Board in compliance with all applicable federal regulations governing the protection of human subjects. Research data were derived from approved Naval Health Research Center Institutional Review Board protocol number NHRC.2020.0008.

## Abbreviations

The following abbreviations are used in this manuscript:

BCa	Bias-corrected and accelerated
BIA	Bioelectrical impedance analysis
BMI	Body mass index
EB	Energy balance
EE	Energy expenditure
EI	Energy intake
FFM	Fat-free mass
FM	Fat mass
GLM	General linear model
IQR	Interquartile range
MRE	Meal, Ready-to-Eat
MSKI	Musculoskeletal injury
POI	Program of instruction
SD	Standard deviation
SE	Standard error
SMM	Skeletal muscle mass
USMC	United States Marine Corps
