# Supplementary material for: The Association Between Energy Balance and Body Composition Changes in Recruits During Marine Corps Basic Training
Source: Nutrients. 2026 Oct 14;18(20):3363. doi: 10.3390/nu18203363 (PMC13708446; doi:10.3390/nu18203363)
Supplement: Supplementary file 1 [file nutrients-18-03363-s001.zip › nutrients-4567359-supplementary.pdf]

**Supplementary Materials for:** The Association Between Energy Balance and Body Composition Changes in Recruits During Marine Corps Basic Training

**Authors:** Jordan Beall, Andrea Givens, Thi Ngo, Emily Kloss, Brenda Niederberger, Laura Palombo, Daniel Bennett, and Karen R. Kelly

**Table S1**. Baseline characteristics and body composition changes by number of valid energy balance days

| Characteristic | 1 day, n=1 | 2 days, n=5 | 3–5 days, n=41 | 6–8 days, n=92 | 9–10 days, n=19 | *p*-value |
|---|---|---|---|---|---|---|
| Age, y | 19 | 18.0 ± 1.0 | 18.7 ± 1.6 | 19.0 ± 1.6 | 19.1 ± 1.6 | 0.414 |
| Baseline body mass, kg | 65.9 | 60.7 ± 6.4 | 63.6 ± 10.5 | 64.1 ± 10.6 | 67.6 ± 11.5 | 0.453 |
| Baseline FM, kg | 21.6 | 14.4 ± 5.2 | 13.8 ± 6.1 | 14.1 ± 6.0 | 12.5 ± 5.8 | 0.758 |
| Baseline body fat, % | 32.8 | 23.3 ± 7.1 | 22.0 ± 9.4 | 22.0 ± 8.5 | 18.5 ± 7.9 | 0.400 |
| Baseline SMM, kg | 24.5 | 25.4 ± 2.6 | 27.7 ± 7.0 | 27.7 ± 6.2 | 30.9 ± 6.4 | 0.182 |
| FM change, kg | -2.9 | -2.6 ± 2.6 | -3.6 ± 2.7 | -2.8 ± 2.8 | -2.2 ± 3.0 | 0.297 |
| SMM change, kg | 0.1 | 1.0 ± 0.8 | 0.9 ± 1.3 | 1.0 ± 1.3 | 1.1 ± 1.4 | 0.948 |

*Note.* FM = fat mass; SMM = skeletal muscle mass. Values are mean ± SD, except for the 1 valid day category ($n$ = 1), for which the individual value is shown. The 1-day category was retained for descriptive reporting but excluded from inferential comparisons because within-group variability could not be estimated. $p$-values are from exploratory one-way ANOVA comparing participants with 2, 3–5, 6–8, and 9–10 valid energy balance days. For female baseline body weight and baseline fat mass (kg), n = 105.

**Table S2.** Pre- and Post-Training Body Composition Changes Stratified by Sex

| Outcome | Sex | Baseline | Post-training | Absolute change (95% CI) | Relative change, % (95% CI) |
|---|---|---|---|---|---|
| Body mass, kg | Female | 60.7 ± 8.5 | 59.8 ± 7.7 | -0.89 (-1.53, -0.24) | -1.15 (-2.23, -0.06) |
| | Male | 71.6 ± 10.6 | 70.2 ± 9.1 | -1.33 (-2.43, -0.24) | -1.48 (-2.95, -0.01) |
| Fat mass, kg | Female | 16.0 ± 5.3 | 12.8 ± 4.2 | -3.26 (-3.80, -2.73) | -18.83 (-21.55, -16.10) |
| | Male | 9.5 ± 4.6 | 7.2 ± 3.2 | -2.24 (-3.03, -1.46) | -19.06 (-25.42, -12.70) |
| Body fat, % | Female | 26.0 ± 6.5 | 21.1 ± 5.5 | -4.88 (-5.53, -4.23) | -17.98 (-20.25, -15.70) |
| | Male | 12.9 ± 4.9 | 10.1 ± 3.8 | -2.74 (-3.57, -1.91) | -18.58 (-24.07, -13.09) |
| Skeletal muscle mass, kg | Female | 24.4 ± 3.4 | 25.8 ± 3.5 | +1.39 (+1.17, +1.61) | +5.76 (+4.84, +6.69) |
| | Male | 35.2 ± 4.7 | 35.5 ± 4.4 | +0.26 (-0.09, +0.61) | +0.93 (-0.10, +1.95) |

Note. Values represent Mean ± SD for Baseline and Post-training, and Mean (95% Confidence Interval) for Absolute and Relative changes. Absolute change was calculated as post-training minus baseline; negative values indicate decreases. Relative percentage change was calculated as (post-training − baseline)/baseline×100. Body-fat percentage is reported as a percentage; its absolute change is expressed in percentage points. Confidence intervals are 95% CIs for mean change. For female baseline body weight and baseline fat mass (kg), n = 105. These sex-stratified values are presented descriptively to provide context for the present energy balance analyses. Detailed pre – post body composition analyses from this recruit cohort have been reported previously by Kloss et al.

**Table S3**. Program of Instruction for Energy Balance Assessment Days

| Training Week | Training Day | Training Event | Meals | Snack |
|---|---|---|---|---|
| 1 | 1 | 4x400m intervals, classroom instruction | Mess Hall | No |
| | 3 | Circuit training, combat care class | Mess Hall | No |
| 4 | 19 | Confidence course, injury prevention class | Mess Hall | Yes |
| | 21 | Combat water survival, confidence course | Mess Hall | Yes |
| 7 | 37 | 8k hike | B, L: MRE; D: Box | Yes |
| | 39 | Combat Fitness Test | B, D: Mess Hall; L: Box | Yes |
| 10 | 54 | Crucible 6k hike | MRE | No |
| | 55 | Crucible | MRE | No |
| | 56 | Crucible 15k hike | MRE | No |
| | 57 | Gear turn-in | Mess Hall | No |

*Note.* B = breakfast, L = lunch, D = dinner.

**Table S4.** Sex-stratified adjusted associations between estimated energy balance during sampled days and change in skeletal muscle mass.

| Sex | *B* | BCa 95% CI | Bootstrap *p*-value | $R^2$ | Adjusted $R^2$ |
|---|---|---|---|---|---|
| Male | 0.000248 | 0.000001, 0.001 | .033 | .556 | .508 |
| Female | 0.000119 | -0.000019, 0.001 | .285 | .108 | .063 |

*Note*. CI = confidence interval. The dependent variable was change in skeletal muscle mass (kg) from baseline to post-training. Sex-stratified models were adjusted for cohort, baseline body weight, and number of valid 7-day energy balance assessment days. BCa 95% CIs and p-values were based on 2,000 bootstrap resamples. These sex-stratified models are presented to describe within-sex associations; the pooled sex × energy balance interaction was the formal test of whether energy balance and skeletal muscle mass slopes differed by sex.

**Table S5.** Summary of sensitivity analyses replacing baseline body weight with baseline fat mass or skeletal muscle mass

| **Outcome** | **EB window** | **N** | **EB slope *B* (BCa 95% CI)** | **Bootstrap *p*** | **Male sex × EB *B* (BCa 95% CI)** | **Bootstrap *p*** |
|---|---|---|---|---|---|---|
| FM | 10-day | 157 | 0.00082 (0.00042, 0.00132) | < .001 | Not fitted | N/A |
| FM | 7-day | 157 | 0.00118 (0.00076, 0.00157) | < .001 | Not fitted | N/A |
| SMM | 7-day | 157 | 0.00038 (0.00007, 0.00067) | 0.021 | 0.00064 (0.00007, 0.00122) | 0.019 |
| SMM | 10-day | 157 | 0.0005 (0.00023, 0.00079) | 0.002 | −0.000001 (−0.00037, 0.00038) | 0.994 |

*Note.* FM = fat mass; SMM = skeletal muscle mass; EB = estimated energy balance (kcal/day); BCa = bias-corrected and accelerated; CI = confidence interval; N/A = not applicable. Baseline FM replaces baseline body weight for FM models; baseline SMM replaces baseline body weight for SMM models. All analyses use the same 157 participants as the baseline body weight models, without a valid day restriction. Models also include sex, categorical training cohort, estimated EB, and valid EB days within the corresponding window. FM models omit sex × EB; SMM models retain it. The FM EB coefficient is the common adjusted slope across sexes. The SMM EB coefficient is the female-specific slope; the male sex × EB coefficient is the male–female difference in slopes. Female and Cohort 3 are the reference categories. Outcomes are baseline-

to-post-training changes in kg; *B* values are unstandardized estimates. Two-sided bootstrap *p*-values and BCa 95% CIs use 2,000 resamples. Separately fitted baseline FM interaction models were nonsignificant (10-day bootstrap $p = .517$; 7-day bootstrap $p = .891$).

**Table S6.** Summary of sensitivity analyses excluding participants with two or fewer valid energy balance days within the corresponding sample day window

| Outcome | EB window | N | EB slope B (BCa 95% CI) | Bootstrap p | Male sex × EB B (BCa 95% CI) | Bootstrap p |
|---|---|---|---|---|---|---|
| FM | 10-day | 151 | 0.00126 (0.00074, 0.00182) | < .001 | Not fitted | N/A |
| FM | 7-day | 129 | 0.001786 (0.00127, 0.00233) | < .001 | Not fitted | N/A |
| SMM | 7-day | 129 | 0.00027 (−0.000211, 0.00075) | 0.242 | 0.00065 (−0.000034, 0.00139) | 0.066 |
| SMM | 10-day | 151 | 0.000413 (0.00008, 0.00076) | 0.015 | 0.000044 (−0.00038, 0.00048) | 0.835 |

*Note.* FM = fat mass; SMM = skeletal muscle mass; EB = estimated energy balance (kcal/day); BCa = bias-corrected and accelerated; CI = confidence interval; N/A = not applicable. Participants were required to have at least three valid days within the window being modeled. Models retain baseline body weight, sex, categorical training cohort, estimated EB, and valid EB days as predictors. FM models omit sex × EB; SMM models retain it. Complete-case samples comprise 151 participants (52 males, 99 females) for the 10-day models and 129 (48 males, 81 females) for the 7-day models. The FM EB coefficient is the common adjusted slope across sexes. The SMM EB coefficient is the female-specific slope; the male sex × EB coefficient is the male–female difference in slopes. Female and Cohort 3 are the reference categories. Outcomes are baseline-to-post-training changes in kg; *B* values are unstandardized estimates. Two-sided bootstrap *p*-values and BCa 95% CIs use 2,000 resamples.
